# Analysis of voice quality in patients with late-onset Pompe disease

**DOI:** 10.1186/s13023-016-0480-5

**Published:** 2016-07-15

**Authors:** Krzysztof Szklanny, Ryszard Gubrynowicz, Katarzyna Iwanicka-Pronicka, Anna Tylki-Szymańska

**Affiliations:** Multimedia Department, Polish-Japanese Academy of Information Technology, Warsaw, Poland; Department of Audiology and Phoniatrics, The Children’s Memorial Health Institute, Warsaw, Poland; Department of Paediatrics, Nutrition and Metabolic Diseases, The Children’s Memorial Health Institute, Warsaw, Poland

**Keywords:** Pompe disease, Metabolic disorders, Genetic disorder, Myopathy, Voice quality, Electroglottography, Acoustic methods, Vocal folds, Nasalance measurement, Voice disorders

## Abstract

**Background:**

Pompe disease is a progressive metabolic myopathy. Disease progression is characterized, among other features, by progressive dysfunction of the voice apparatus. The aim of this study was to employ electroglottographic, acoustic and nasalance measurement methods on patients with late-onset Pompe disease in order to provide detailed information on the effect of the disease on voice quality. Voice quality is the key factor for estimating the effectiveness of ERT in late-onset Pompe disease. The study compared clinical phoniatric examination with electroglottographic, acoustic and nasalance measurement methods. The consistency of the aforementioned analyses was assessed.

**Methods:**

The study examined 19 patients with late-onset Pompe disease (including 9 with the juvenile form of the disease). Of these, a total of 17 patients underwent otolaryngological examination with detailed phoniatric evaluation of their articulatory organs. Electroglottographic recordings and nasalance measurements (using the nasalance Separator Handle) were obtained from all patients. MATLAB (COVAREP toolkit) was used to analyse voice recording data.

**Results:**

Dysphonia observed in patients with late-onset Pompe disease is mainly caused by dysfunction of vocal fold closure and weakness of vocal muscle. However, substantial speech nasality is caused by insufficient closure of the soft palate. Electroglottographic signal analysis, acoustic and nasalance testing methods indicated that more significant changes in the function of the voice apparatus presented in the juvenile form than in the adult form of late-onset Pompe disease.

**Conclusions:**

It was found that speech nasality and electroglottographic tests are more repeatable, comparable and versatile than phoniatric examination, allowing for earlier detection of voice pathology in late-onset Pompe disease. These sensitive and non-invasive acoustic and electroglottographic methods allow for the tracking of changes in voice as patients undergo treatment or as the disease progresses.

## Background

Pompe disease (glycogen storage disease type II, GSD II) is a progressive metabolic myopathy caused by a deficiency of the lysosomal acid alpha-glucosidase. This enzyme deficiency leads to an accumulation of glycogen, mainly in the muscles, resulting in their progressive destruction [1, 2]. The spectrum of clinical phenotypes includes an infantile form (classic form) and a late-onset form (with both juvenile and adult presentations). In the juvenile form (late-onset) the first symptoms, such as progressive proximal and axial muscle weakness, appear between 2–5 years of age. The adult form (late-onset) has a slower progression, with the first symptoms appearing in adulthood. Late-onset clinical features include progressive muscle weakness, with particularly damaging effects in respiratory muscles, necessitating ventilator-assisted breathing in advanced stages [3]. With disease progression, the effects of muscle cell damage and destruction display clearer clinical manifestations, with abnormalities developing in the voice apparatus.

Patients with advanced late-onset Pompe disease experience speech disorders in all forms of the disease [3–6]. In addition Hobson-Webb et al. [5] described the presence of articulation disorders and dysarthria in late-onset Pompe disease patients. Jones et al. revealed lingual weakness to be present in 80 % of subjects with late-onset Pompe disease [6]. Papers published to date have not employed electroglottographic, acoustic nor nasalance testing methods in the clinical assessment of late-onset Pompe disease. However, these investigative methods have been successfully used to study voice disorders [7–9] and are widely available, inexpensive and non-invasive. The authors wished to study whether these methods could be applied to assess voice quality and thereby measure the effectiveness of ERT in late-onset Pompe disease on the functioning of the voice apparatus. These methods are automated and possess the advantages of objectivity, repeatability and comparability. According to Jones et al. [6] it is important to find methods that increase suspicion of late-onset Pompe disease.

## Methods and patients

The study and its consent procedure were approved by the Bioethics Committee (133/KBE/2014) of the Children’s Memorial Health Institute in Warsaw. All study subjects gave informed, written consent prior to their participation; consent on behalf of all children taking part was given in writing by their parents or guardians. The study examined 19 patients with late-onset Pompe disease, from 14 families, ranging in age from 7 to 54. The mean age of patients at the time the study was performed was 28.2 years, with a median of 39. 9 patients had the juvenile form (group 1) and 10 had the adult form (group 2) of the disease. All patients were on ERT at the moment of investigation. The therapy lasted from 3 to 8 years. Patients’ clinical data, mutation and length of ERT are shown in Table 1.

**Table 1:** Patient demographics

ID	Gender	Current age years	Age of first symptoms years	Age of diagnosis years	Years on ERT	Mutation	Form
1	F	11.8	no symptoms, family screening	2	7	IVS1-13T>G/c.2662G>T	Juv
2	M	15.5	no symptoms, family screening	6	7	IVS1-13T>G/c.2662G>T	Juv
3	F	17.9	2	2.5	7	IVS1-13T>G/c.307T>G	Juv
4	M	25.6	6	15	7	IVS1-13T>G/c.2662G>T	Juv
5	F	8.3	0.5	2	6	L291F, 871C>T/R600C, 1798C>T	Juv
6	F	7.5	1	1.5	6.5	2495delCA (ex18)/2495delCA (ex18)	Juv
7	M	8.5	no symptoms, family screening	0.6	6	G377S c.2495_2496 delCA	Juv
8	F	14.8	3	4	8	C1129G>A/c.2495_2496 delCA	Juv
9	M	17.8	3.5	4	7	IVS1-13T>G/c.925G>A	Juv
10	F	40	6	31	7	c.364A>G/c.1796C>T	Adult
11	F	31	7	25	7	IVS1-13T>/C103G	Adult
12	M	37.5	27	29	7	c.364A>G/c.1796C>T	Adult
13	F	39	25	34	3	IVS1-13T>G/C103G, 307T>G	Adult
14	M	46.5	35	40	7	IVS1-13T>G/c.307T>G	Adult
15	F	53.8	30	46	5.5	IVS1-13T>G/525delT	Adult
16	M	34.8	15	25	7	IVS1-13T>G/c.307T>G	Adult
17	M	53.8	33	48	8	IVS1-13T>G/C103G, 307T>G	Adult
18	M	37.8	28	32	5	IVS1-13T>G/c.307T>G	Adult
19	F	33.8	26	30	5	IVS1-13T>G/c.307T>G	Adult

Table 1. Juv - juvenile

Patients were invited to participate in a phoniatric evaluation of their voice apparatus, alongside electroglottographic, acoustic and nasalance measurement methods of evaluation.

### Phoniatric evaluation of the voice apparatus

A phoniatric examination was performed on 17 out of the 19 patients, including an assessment of ears, nose, oral cavity, nasopharynx, middle and lower oropharynx and larynx. The study was supplemented by voice quality assessment based on perceptual evaluation of voice quality on the GRBAS scale [10, 11]. Phoniatric examination of the condition of the vocal tract was carried out with an endoscopic set (video-otoscope 0.6 mm, flexible video-fiberscope 2.5 mm, 90-degree Hopkins video-laryngoscope) and a Carl Zeiss ear microscope. The breathing pattern of each patient was evaluated by observing chest and neck movements, how the voice was created, phonatory and breathing coordination, and phonation time. Nasalance assessment was conducted using Czermak’s mirror test (mirror-fogging test) of nasal air escape.

### Acoustic method of voice quality analysis

Nineteen patients with late-onset Pompe disease participated in acoustic and electroglottographic recordings. Equipment from Glottal Enterprises, a Nasalance Separator Handle and an EG2-PCX2 electroglottograph with microphone were used in the study. The noise signal was reduced by 40 dB in the acoustic signal as well as in the electroglottographic signal.

### Electroglottography

An analysis of the electroglottographic signal has been shown to correlate best with various types of voice quality. In comparison to modal phonations, it is possible to differentiate between a breathy voice, a creaky voice and a tense voice. EGG was used to detect vocal fold vibration pattern [7, 12, 13].

To carry out the analysis, the parameters CQ H (Closing Quotient) and SQ (Speed Quotient) were calculated. CQ H measures the duration of the closing phase of the glottal cycle and is a hybrid calculation, using the EGG contacting peak for detecting the glottal contact event, and an EGG-based 3/7 threshold for detecting the glottal opening event [14–16]. SQ (Speed Quotient) is the ratio between increased contact during the closing phase duration and opening phase durations of the glottal cycle. It is an expression of the symmetry of glottis air exchange [17].

For EGG recordings, patients phonated the vowel /a/ three times for a sustained period with natural volume. These recordings were used to assess vocal fold vibration and voice quality. MATLAB (COVAREP toolkit) was used for further analysis [18].

### Acoustic analysis

Four parameters (Peak Slope, NAQ, HRF & CPPv) [19, 20, 22, 23] were used to assess voice quality in patients with late-onset Pompe disease. Peak Slope has demonstrated the ability to differentiate between breathy modal and tense voice. The main advantage of the Peak Slope algorithm is that it functions as a standalone program. Normalized Amplitude Quotient (NAQ) has been used in prior studies to assess tense voice.

Both parameters can be applied, even in recordings with background noise. The experiments demonstrated, among other things, that applying the NAQ parameter to calculations on real speech signals allows for differentiation between normal, breathy and pressed phonations [19–21]. Employing the Harmonic Richness Factor (HRF) parameter permits the detection of dysphonia [22] and the Cepstral Peak Prominence parameter allows detection of early dysphonia. The efficacy of the CPPv method has been validated in prior work by Hillenbrand et al., and Maryn et al., [23, 24]. The occurrence of subharmonics in the EGG signal was examined by Praat [25, 26].

For the purposes of the acoustic analysis, the microphone signal obtained in the EGG recordings was used.

### Nasalance measurements

The Nasalance Separator Handle (Glottal Enterprises), a computer-assisted instrument similar to a nasometer [27], was used in this study for tracking variations in nasalance (the acoustic correlate of perceived nasality), which is the ratio of nasal over nasal plus oral acoustic energy during speech. The value of this coefficient depends strongly on the nasal surface of the velum, which in the case of nasal speech is relatively large [28].

Hajja [29] demonstrated that acoustic examination of nasality changes is efficient for tracking the progress of nasal speech rehabilitation.

Nasalance recording of the following sounds was carried out: sequences of the Polish vowels /y/ /e/ /a/ /o/ /u/ /i/; voiced plosives separated by vowels (sequence type: V-VP-V-VP...); and nasal consonants separated by vowels and the sustained vowel /i/. The entire recording was used to assess nasality.

## Results

**Fig. 1:**
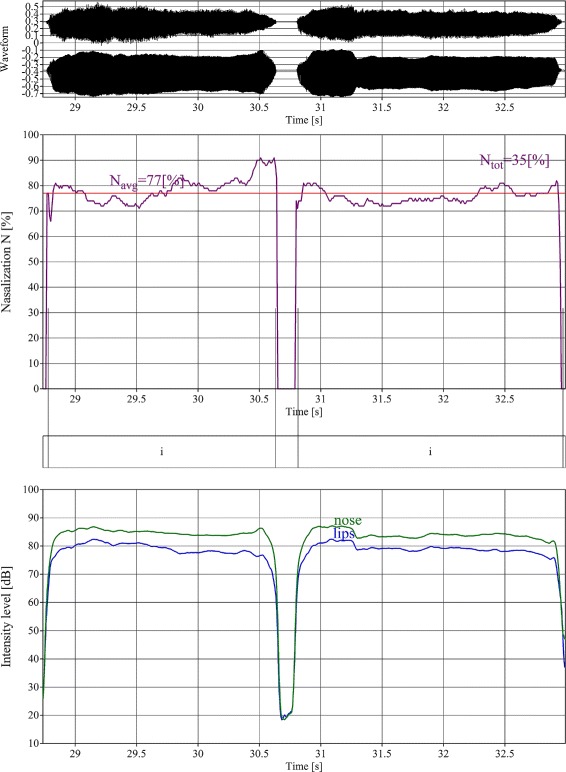
Nasalization analysis of two sustained vowel sounds /i/ articulated by patients with late-onset Pompe disease (group 1). Two time signals (top), measurement of the nasalization coefficient (central) and time level variations in dB (bottom) of nose (continuous line) and mouth (broken line) signals. In healthy children’s voices, this coefficient does not exceed 20 %. This is typical for hypernasal speech

A summary of the test results is presented in Tables 1, 2, 3, 4 and Fig 1. Tables 2 and 3 show the results of the phoniatric examination, including the assessments of the oral cavity, ears, nose and larynx. Table 4 shows the results of the acoustic and electroglottographic analyses. Figure 1 shows the result of hypernasal speech.

**Table 2:** Laryngological examination results in patients with late-onset Pompe disease

ID	Czermak score	Oral cavity	Nose	Ears
1	ND	N	DSN	N
2	ND	N	N	N
3	2	SP atonic, LMPh	N	N
4	ND	SP atonic, LMPh	N	N
5	2	Tonsillar hypertrophy, Short SP, Short SP		OMS bil
6	2	SP atonic, short LMPh	N	N
7	2	Short atonic SP LMPh, atonic tongue	Conchal O	N
8	2	Short atonic SP, LMPh, atonic tongue	DSN	N
9	0	N	DSN	Epitympanal retr.
10	1	Short atonic SP, LMPh Lack of pharyngeal reflexes, Geographic tongue, Malocclusion (open bite)	N	N
11	0	N - Normal palate	N	N
12	0	SP LMPh	N	N
13	ND	ND	ND	ND
14	0	Long slender, movable, SP	DSN	N
15	1	N - Normal palate	N	Min. retractions of the drums
16	0	N - Normal palate	N	N
17	0	N - Normal palate Hypertrofia Tonsillae,	N	Osteoma in the right external auditory meatus
18	1	Short SP, PPHI, LMPh	DSN	N
19	1	Short SP, PPHI	Conchal O	Epitympanal retr.

Table 2. SP – soft palate; LMPh – limited mobility during phonation; DSN – deviation of nasal septum; OMS – otitis media secretoria; PPHI – palatopharyngeal insufficiency; retr – retractions; ND – not done

**Table 3:** Video-laryngoscopic examination results in patients with late-onset Pompe disease

ID	Larynx diagnosis	Age of dysphonia/duration	GRBAS	MPT	Vestibulum of larynx	Vocal folds	Arytenoid area
1	N	No	00000	6 s.	N	GI pp, VF thickened, thick mucus	Min. C
2	N	No	00000	8 s.	N	Min. VF thickened	N
3	GI Laryngeal tremor	No data	00100	10 s.	N	GI mp, VF tremor thick mucus on VF	C, O
4	N	No data	10100	4 s.	N	VFC, mucus on VF	N
5	Hyperfunctional dysphonia	No data	00001	10 s.	Phonation of VsP	N	C
6	No data	No data	No data	No data	No data	No data	No data
7	GI	4 H, silent soundless matte V	00220	12 s.	N	GI mp	Min. C, O
8	GI	13/0.1, silent soundless matte V	10110	12 s.	N	GI mp	C, O, mucus retention
9	N	No	00000	20 s.	N	N	N
10	GI Laryngitis posterior	25/10 H	10000	15 s.	N	GI	C, O Laryngitis posterior
11	N	No	00000	15 s	N	N	N
12	GI	30/8 morning H cough	00000	18 s.	N	GI mp	Min. C
13	No data	No data	No data	No data	No data	No data	No data
14	GI	37/10	00110	8 s.	Enlarged rVsF covers rVF	GI Oblique orientation of glottis.	N
15	GI	No V fatigue	00110	11 s.	N	GI mp. VF C	C, O
16	N	No	00100	5 s.	N	VF thickened, tremor	Min. C, O
17	GI	38/4	00100	22 s.	N	GI mp	C, O
18	GI	No V fatigue	10000	5 s.	N	GI mp	Min. C. As. r aryt. Moved forward
19	GI	No data	00100	4 s.	N	GI mp	N

Table 3. GI - Glottal insufficiency; VF – vocal folds; VsF – vestibular folds; C – congestion; O - oedema; mp - middle part of the glottis; Min. – minimal; H – hoarseness; V – voice; r – right; N – normal, MPT –maximum phonation time

**Table 4:** Coefficients for PS, NAQ, HRF, CPPv, CQ H and SQ values were obtained during prolonged phonation of /a/. In addition, the CQ H coefficient was calculated for its fragments

ID	PS	NAQ	HRF	CPPv	CQ H	CQ H 2 sec.	CQ H 1sec.	SQ
1	-0.21	0.11	14.72	11.90	0.45	0.38	0.36	0.28
2	-0.40	0.10	34.20	11.36	0.31	0.27	0.26	0.85
3	-0.22	0.09	13.60	11.81	0.45	0.43	0.42	0.30
4	-0.39	0.09	30.97	10.95	0.36	0.34	0.34	0.60
5	-0.06	0.08	16.10	11.57	0.20	0.18	0.17	1.60
6	-0.12	0.11	12.70	11.36	0.34	0.34	0.31	0.38
7	-0.02	0.16	13.60	11.25	0.30	0.29	0.28	0.28
8	-0.16	0.11	11.90	11.54	0.37	0.32	0.30	0.38
9	-0.50	0.12	37.49	10.31	0.23	0.23	0.23	1.20
10	-0.43	0.19	17.51	11.57	0.29	0.28	0.27	0.84
11	-0.29	0.08	19.20	12.49	0.46	0.45	0.43	0.13
12	-0.26	0.13	17.11	11.46	0.44	0.40	0.37	0.29
13	-0.33	0.14	17	12	0.43	0.39	0.38	0.43
14	-0.48	0.15	21.85	11.93	0.37	0.34	0.33	0.45
15	-0.10	0.12	12.70	11.40	0.39	0.36	0.35	-
16	-0.43	0.11	29.30	11.52	0.24	0.22	0.21	-
17	-0.44	0.18	26.67	11.76	0.32	0.30	0.28	1.08
18	-0.34	0.10	30.64	11.24	0.30	0.28	0.27	-
19	-0.09	0.09	13	11.51	0.35	0.34	0.33	-

### Phoniatric assessment results

In group 1, voice irregularities were observed in all the patients (tense voice type), characterized by excessive muscle tension of the shoulder girdle, neck and submandibular areas. Voice pitch was altered in all the patients in group 1.

Dysphonia was observed in 5 out of 8 patients in group 1. Only 2 patients independently noticed symptoms of hoarseness (indicating dysphonia). Angioedema caused minimal changes to the vocal folds in 3 patients, and an accumulation of mucus presented in 3 patients. Changes in the posterior commissure were observed in 5 patients. Swelling of different intensities was observed in 3 patients, while minor redness was seen in 5 patients. Features of hyperfunction, expressed by vestibular fold phonation, were observed in one patient.

Glottal phonatory insufficiency, being a lack of full vocal fold closure in phonation, was observed in 3 patients. Severe nasality was observed in 5 patients. This resulted from either the soft palate being short, limited palate mobility or both and was also confirmed by the Czermak test (Table 2).

In group 2, phoniatric tests were carried out on 9 out of 10 patients (Table 3). Dysphonia, caused by excessive muscle tension in the shoulder girdle, neck and submandibular areas, was observed in 7 out of 10 patients. Fluctuations in voice pitch and tense voice were observed in these patients.

Voice quality disorders were observed in 6 patients; 3 of them were able to report when their dysphonia first appeared.

Glottal insufficiency was observed in 7 patients using video-laryngoscopic examination, with the middle part of the glottis primarily affected (5 patients). Swelling of the posterior commissure was observed in 4 patients and varying degrees of redness were seen in 5. Asymmetry in laryngeal structures was found in 2 patients and fluctuations in voice pitch in one of them.

Soft nasality was observed in 3 patients. This resulted from either the soft palate being short, limited palate mobility or both and was also confirmed by the Czermak test. Proper functioning of the soft palate was observed in the 6 remaining patients.

### Electroglottographic and acoustic analyses results

In group 1, closing insufficiency of the vocal folds during the whole phonation was observed in 7 out of 9 patients, and in 8 out of 9 patients in a phonation fragment of at least 2 seconds (Table 4 CQ H parameter). Significantly reduced SQ values were observed in 6 patients (mean=0.58, median=0.38, with a range from 0.28 to 1.6).

Clinically proven hyperfunctional dysphonia was found in one patient, where an SQ value of 1.6 was measured. These values differ significantly from normal values when using similar methods [30]. Irregularity in the function of the vocal folds was observed in 5 patients. Nonsynchronous movement of vocal folds was observed in 1 patient.

Symptoms of dysphonia were observed in 7 patients and tense voice was observed in 8 patients (Table 4 parameters PS, NAQ, HRF & CPPv).

In group 2, glottal insufficiency was detected in 7 patients during the entire phonation and in 9 patients during at least 1 second of the phonation of the vowel /a/. The SQ value was observed to be significantly reduced in 4 patients (mean=0.63, median=0.44, with a range from 0.13 to 0.84) [30]. Irregularity in the function of the vocal folds was observed in 5 patients. Nonsynchronous movement of the vocal folds was observed in 3 patients.

Symptoms of dysphonia were observed in 7 patients, and tense voice was observed in the other 7 patients with the adult form of the disease (Table 4 parameters PS, NAQ, HRF, CPPv).

### Nasalance measurement results

In group 1, significant nasality was observed in 5 patients, and open nasality was observed in 2 patients. Velopharyngeal closure insufficiency was detected in 7 patients, and limited palate mobility in 1 patient. Significant speech nasality occurred in the same patients in whom vocal fold insufficiency was found.

In group 2, soft nasality was observed in 5 patients and limited movement of the soft palate and velopharyngeal closure impairment was seen in the other 4 patients.

## Discussion

Progressive muscle damage in late-onset Pompe disease leads to changes in the voice and speech. A number of speech studies have evidenced articulation disorders and dysarthria, as reported in Dubrovsky et al, Fuller et al, Hobson-Webb et al, and Jones et al. [3–6].

Early onset of symptoms with rapid progression is classified as the juvenile form of the disease and its outcome is more severe. Patients with symptoms appearing later are classified as having adult form [1, 2].

The study compared the usefulness and efficacy of voice quality assessment by clinical phoniatric examination with electroglottographic, acoustic and nasalance measurement methods. The results obtained by all the different methods showed a close degree of compliance. However, acoustic methods showed higher sensitivity, objectivity and reproducibility of results in the studied patients. The parameters obtained in both phoniatric and acoustic analyses of both groups of patients with late-onset Pompe disease indicated significantly more pronounced symptoms in juvenile forms in comparison with adult forms.

Electroglottographic and acoustic analyses evidenced vocal fold insufficiency in both groups, as a consequence of the weakening of the voice muscles. This was consistent with the laryngological assessment. The applied signal parameterization and parameter calculation of the source signal allowed for a more detailed analysis and observation of closure insufficiency in more patients than with video-laryngoscopic examination.

In group 1, EGG the CQ H parameter indicated closing insufficiency in 8 patients in a phonation fragment of at least 2 seconds, compared to 3 patients in the phoniatric assessment.

In group 2, EGG the CQ H parameter detected glottal insufficiency in 7 patients during the entire phonation and in 9 patients for at least 1 second, compared to 7 patients in the phoniatric assessment.

Data inconsistency was observed only in the case of a single patient. This was likely an effect of the time difference between the performance of the phoniatric examination and the electroglottographic recording. The laryngoscopic study was performed four years previously.

The EEG analysis confirmed the irregular ratio of increased contact during the closing and opening phases of the glottal cycle, indicating abnormalities in the function of the laryngeal muscles.

The acoustic analysis parameters [19, 21] in the juvenile as well as in the adult form of the disease provided further evidence of a voice quality shift towards tense voice as a result of respiratory muscle weakness. Voice pitch fluctuation and variation of voice within the same phonation, were also observed. Short-term measurements of electroglottographic signals confirm this observation (Table 4).

In group 1, the Peak Slope parameter indicated breathy voice phonation in 6 patients and tense voice in 3 patients in the whole phonation. The NAQ parameter indicated tense voice in 8 patients. The CPPv parameter indicated dysphonia in 7 patients in the whole phonation. The HRF parameter indicated dysphonia in 6 patients. The phoniatric assessment found dysphonia in 5 patients.

In group 2, the Peak Slope parameter indicated breathy voice phonation in 2 patients and tense voice in 5 patients in the whole phonation. The NAQ parameter indicated tense voice in 7 patients. The CPPv parameter indicated dysphonia in 7 patients in the whole phonation. The HRF parameter indicated dysphonia in 5 patients. The phoniatric assessment found dysphonia in 7 patients. The Peak Slope parameter had not been used for voice analysis until this study [19].

In group 1, a subharmonic vibratory pattern produced in the larynx, functioning with half of the fundamental frequency (F0), was observed in 1 patient, and with group 2 in 3 patients.

It is noteworthy that the specification of the Peak Slope and NAQ parameters gave the opportunity to create a system for self-assessment of voice quality by a patient in the form of an application on his smartphone.

Phoniatric examination showed a short soft palate. In the juvenile form of late-onset Pompe disease, the process of muscle tissue atrophy is faster than in the adult form of late-onset Pompe disease. Therefore, the dysfunction of the voice apparatus is clearly marked. In the adult form, symptoms are less severe, and correlate with the weakness of other muscle groups. The Czermak test showed consistency with the nasality analysis. However, an acoustic analysis allows for more accurate determination of the degree of nasality and velum malfunction.

For the late-onset form of Pompe disease, an acoustic analysis was not carried out. The obtained results permitted an assessment of the dynamics of disease progression and treatment effects. The applied methods offer greater accuracy and reproducibility in the analysis of vocal fold function and allow an assessment of the type of phonation. Electroglottographic analysis was the most sensitive of all the methods used.

Since devices for performing acoustic measurements are not expensive, it is possible to develop a system allowing the patient to control the quality of voice with two parameters, PS and NAQ. In carrying out acoustic recordings, special acoustic conditions are not required, as has been proven by Kane [19].

The presented methods permit an evaluation of some voice features, as well as nasality and vocal fold functioning. Furthermore, the ability to perform both long and short-term analyses allows the tracking of discrete changes in the vocal folds, which is undetectable with video-laryngoscopy assessment.

## Conclusions

Electroglottographic, acoustic and nasalance measurement methods all proved to be more sensitive, repeatable, comparable and versatile than phoniatric examination. These methods are suitable for assessing voice quality and allow an evaluation of voice impairment in patients with late-onset Pompe disease.

The PS (Peak Slope) and NAQ (Normalized Amplitude Quotient) parameters allow the evaluation and tracking of voice changes in the patient under normal acoustic conditions.

The relatively low cost of the study, the ease of data retention and the reliability of the three analysis methods are significant advantages that could be exploited to increase the efficiency of tracking the dynamics of late-onset Pompe disease progression.

This study explored the range of pathological changes in voice in patients with late-onset Pompe disease, and the varying degrees in severity of these changes.

## Abbreviations

COVAREP, a cooperative voice analysis repository for speech technologies; CPP, cepstral peak prominence; CQ H, closing quotient; EGG, electroglottography; ERT, enzyme replacement therapy; F0, fundamental frequency; HRF, harmonic richness factor; GRBAS, grade, roughness, breathiness, asthenia, strain scale; GSD II, glycogen storage disease type II; NAQ, normalized amplitude quotient; PS, peak slope; SQ, speed quotient, V, Vowel; VP, voiced plosives separated by vowels
